# Prognostic Value of Lung Ultrasound in Aortic Stenosis

**DOI:** 10.3389/fphys.2022.838479

**Published:** 2022-04-05

**Authors:** István Adorján Szabó, Luna Gargani, Blanka Morvai-Illés, Nóra Polestyuk-Németh, Attila Frigy, Albert Varga, Gergely Ágoston

**Affiliations:** ^1^ GE Palade University of Medicine, Pharmacy, Science and Technology of Tîrgu Mure, Tîrgu Mure, Romania; ^2^ Department of Surgical, Medical and Molecular Pathology and Critical Care Medicine, University of Pisa, Pisa, Italy; ^3^ Department of Family Medicine, University of Szeged, Szeged, Hungary

**Keywords:** aortic stenosis, heart failure, pulmonary congestion, lung ultrasound, prognosis

## Abstract

**Background:** Aortic stenosis (AS) is the most common primary valve lesion requiring intervention in Europe and North America. It has a prolonged subclinical period during which, as AS worsens, left ventricular adaptation becomes inadequate and impaired systolic and/or diastolic dysfunction may lead to overt heart failure (HF). The development of HF is an inflexion point in the natural history of AS. Pulmonary congestion is a cardinal feature in HF, and lung ultrasound (LUS) evaluation of B-lines has been proposed as a simple, noninvasive tool to assess pulmonary congestion.

**Aim:** To assess the presence and the prognostic value of sonographic pulmonary congestion in patients with moderate or severe AS.

**Methods:** 75 consecutive patients (39 women, mean age 73.85 ± 7.7 years) with moderate or severe AS were enrolled. All patients underwent comprehensive echocardiography and LUS with the 28 scanning-site assessment. Patients were followed-up for 13.4 ± 6 months to establish the prognostic value of LUS. A composite endpoint of death (of any cause), hospitalization for HF and intensification of loop diuretic therapy was considered.

**Results:** We found a severe degree of B-lines (≥30) in 29.33% of patients. The number of B-lines correlated with the estimated pulmonary artery systolic pressure (*p* < 0.001, r = 0.574) and increased along with NYHA class (*p* < 0.05, rho = 0.383). At multivariable analysis, B-lines ≥30, and mean gradient were the independent predictors of events [B-lines: 2.79 (CI 1.03–7.54), *p* = 0.04; mean gradient: 1.04 (CI 1.01–1.07), *p* = 0.004].

**Conclusion:** Evaluation of B-lines is a simple, highly feasible method to detect pulmonary congestion in AS. The number of B-lines correlates with the hemodynamic changes caused by AS and with the functional status of patients. A severe degree of sonographic pulmonary congestion is associated with an increased risk of adverse events.

## Introduction

Aortic stenosis (AS) is the most frequent degenerative valvular heart disease in Western countries; its prevalence continuously increases with ageing (Iung et al., 2003; Nkomo et al., 2006; Vahanian et al., 2021). The development of heart failure (HF) symptoms is a determinant factor in the survival of patients with AS (Frank et al., 1973). The correlation between the severity of AS and the onset of symptoms is poor and depends mostly on the hypertrophic, compensatory response of the left ventricle (LV) to pressure overload (Vahanian et al., 2021). LV hypertrophy is a compensatory mechanism to restore wall stress and maintain cardiac output under increasing pressure overload caused by the stenosis. However, progressive loss of cardiomyocytes and myocardial fibrosis that accompanies LV hypertrophy may eventually lead to LV dysfunction. Increased wall thickness also impairs diastolic function and leads to increased filling pressures to achieve the same diastolic volume (Hess et al., 1984). This augmented diastolic pressure leads to pulmonary congestion (PC) and dyspnoea. In more advanced stages of the disease, the pressure overload cannot be counterbalanced by LV hypertrophy, and reduced left ventricular ejection fraction (LVEF) can develop. Decreased LVEF also contributes to the PC and HF symptoms and is associated with poor outcomes (Carabello and Paulus, 2009; Pibarot and Dumesnil, 2012). PC is a frequent and almost universal pathophysiological phenomenon in patients with heart failure. Lung ultrasound (LUS) evaluation of B-lines has been proposed as a simple, noninvasive, radiation-free and semi-quantitative tool to assess PC (Gargani, 2011; Volpicelli et al., 2012; Pellicori et al., 2019). B-lines have been closely linked to the amount of extravascular lung water and pulmonary capillary wedge pressure in HF patients (Agricola et al., 2005; Gargani et al., 2008; Miglioranza et al., 2013). LUS can identify clinically silent pulmonary oedema (Miglioranza et al., 2013), suggesting that it can be utilized to assess hemodynamics and optimize treatment (Pellicori et al., 2019). Our study aimed to determine the prognostic value of LUS B-lines in predicting adverse events in patients with moderate or severe aortic stenosis.

## Methods

Seventy-five consecutive patients with AS from two sites (University Of Szeged, Hungary, Clinical County Hospital Târgu Mures, Romania) were enrolled. The inclusion criteria were: 1) moderate degenerative AS with mean gradient of 20-40 mmHg and aortic valve area (AVA) 1–1.5 cm^2^; 2) or severe degenerative AS with mean gradient >40 mmHg and AVA <1 cm^2^; 3) age >18 years; 4) informed consent. We enrolled patients with severe symptomatic AS, only if the patient refused surgery or it was contraindicated.

The exclusion criteria were: 1) low flow-low gradient AS (mean gradient <40 mmHg, AVA <1 cm^2^, LVEF<50%); 2) concomitant moderate or severe aortic regurgitation; 3) concomitant moderate or severe mitral regurgitation; 4) severe, decompensated HF, requiring urgent hospitalization (NYHA class IV); 5) severe interstitial lung disease; 6) active pneumonia or acute lung injury; 7) malignancy (except localized skin basal cell carcinoma or localized prostatic cancer); 8) cardiomyopathies—dilated, hypertrophic or infiltrative cardiomyopathy. All patients were evaluated in ambulatory settings in rather stable conditions. None of the patients required hospitalization at the time of TTE and LUS. The patients signed informed consent before inclusion in the study. Data handling and publication respected the Declaration of Helsinki. The registration number of ethical approval is 131/2019/SZTE.

A comprehensive transthoracic echocardiogram (TTE) was performed in both sites, using a Vivid-S70 (GE Vingmed, Horten, Norway) ultrasound machine equipped with the 3S probe (1.5–3.6 MHz). Experienced cardiologists, certified by the European Association of Cardiovascular Imaging (EACVI) for TTE, performed all measurements according to the American Society of Echocardiography and EACVI recommendations (Lang et al., 2015; Baumgartner et al., 2017). Longitudinal myocardial strain was analyzed with GE EchoPAC (version v202) software. LV strain was measured according to EACVI recommendations (Lang et al., 2015). The QRS complex was used as a time reference. LA strain parameters were recorded as per the EACVI consensus document and were post hoc analyzed (Badano et al., 2018). ECG trigger was used as a time reference, using the upslope of the R wave as a surrogate of end-diastole.

### Lung Ultrasound

Immediately after TTE, patients underwent B-lines assessment, using the same probe and machine, with the same setting. We screened the anterior and lateral hemithorax, scanning along the parasternal, midclavicular, anterior axillary and mid-axillary lines from the second to the fifth intercostal space on the right hemithorax and from the second to the fourth intercostal space on the left; a total of 28 scanning sites were assessed as previously described (Gargani and Volpicelli, 2014). A B-line was defined as a discrete comet-like vertical hyperechoic reverberation artefact starting from the pleural line, extending to the bottom of the screen and moving synchronously with lung sliding (Volpicelli et al., 2012). The total number of B-lines on the 28 scanned sites (0–10 for each site) was recorded, generating a B-lines score. In each scanning site, the number of B-lines was quantified real-time: when B-lines were distinguishable, they were counted one by one (0–10 for each site); when they were confluent, the percentage of the white screen occupied by B-lines below the pleural line was considered, and then divided by 10 (Volpicelli et al., 2012) (Gargani and Volpicelli, 2014). A total score of B-lines ≥ 30 was considered a cut-off for severe PC (Gargani et al., 2008).

### Follow Up Data

Follow-up data were collected every 3 months *via* phone calls to monitor clinical status and adverse outcomes. Outpatient visits were performed six-monthly, and clinical status, adverse events were recorded. We considered a composite endpoint of events. The endpoint was determined by the following events: death (any cause), HF event requiring hospitalization, and ambulatory intensification of loop diuretic therapy. Data collection was based on a standardized clinical questionnaire performed by a researcher blinded to clinical records. If an endpoint event was detected, details were retrieved from medical records.

### Statistical Analysis

Continuous variables are expressed as mean ± standard deviation or median and interquartile ranges, as appropriate. Two-sample comparisons were performed using the *t*-test and the Chi-square test for categorical data. A *p*-value < 0.05 was set for statistical significance. Correlations between parameters were assessed with parametric Pearson or nonparametric Spearman correlation coefficient analysis, as appropriate. The prognostic performance was determined by means of receiver-operating characteristic (ROC) curves. The association of selected variables with the outcome was assessed by Cox’s proportional hazard model using univariable and multivariable procedures (Backward LR method). We excluded collinearity using variance inflation factor > 3. The event rates were estimated with Kaplan-Meier curves and compared by the log-rank test. Hazard Ratios were reported. Data were analyzed using IBM SPSS 22 statistical software.

## Results

Ninety-seven patients were screened from May 2019 to October 2020: 22 patients were excluded from the initial population (4 patients had concomitant moderate aortic regurgitation, six patients had concomitant moderate or severe mitral regurgitation, four patients had dilated cardiomyopathy with moderate AS, four patients had low-flow, low-gradient AS, three patients had severe chronic obstructive pulmonary disease, and one patient had active lung cancer). Finally, 75 patients (39 women, mean age 73.85 ± 7.7 years) were enrolled in the study. According to the 2021 ESC guideline categorization, the enrolled patient population included 30 patients with high-gradient AS, 22 patients with low-flow, low-gradient AS with a preserved EF, 8 patients with normal-flow, low-gradient AS with preserved EF, and 15 patients with moderate AS. During the 13.4 ± 6 months follow-up, we detected 28 events: 19 patients had hospitalizations due to HF (2 of them underwent urgent AVR), seven of them required ambulatory intensification of loop diuretic therapy. Two patients died (the exact cause of death is unknown). Baseline characteristics of the study population and the comparisons between those with and without events are shown in Table 1.

**TABLE 1:** Clinical characteristics of the study population and comparisons between patients with and without events.

	All Patients (*n* = 75)	Event-free Group (*n* = 47)	HF Event Group (*n* = 28)	*p*
Age (years)	73.85 ± 7.74	72.04 ± 8.1	76.89 ± 6.3	0.008
Gender (female)	39 (52%)	26 (55.3%)	13 (46.4%)	0.456
BMI (kg/m2)	27.11 ± 3.8	26.99 ± 4.19	27.22 ± 3.4	0.812
SBP (mmHg)	127.82 ± 12	126.85 ± 11	129.00 ± 14.7	0.521
DBP (mmHg)	76.66 ± 8	76.71 ± 6	76.71 ± 10.8	0.966
HR (BPM)	70.11 ± 9.4	69.60 ± 9.9	70.43 ± 9.1	0.734
NYHA I	16 (19.2%)	16 (31.4%)	0 (0%)	<0.001
NYHA II	43 (57.3%)	24 (51.0%)	19 (67.8%)	0.185
NYHA III	15 (20%)	6 (12.7%)	9 (32.1%)	0.047
Peripheral oedema	10 (13.3%)	5 (10.6%)	5 (17.8%)	0.374
Syncope	5 (6.67%)	2 (4.2%)	3 (10.7%)	0.302
Rales	12 (16%)	4 (8.5%)	8 (28.5%)	0.048

Data are expressed as mean ± SD, or number and percentage.

BMI, body mass index; SBP, systolic blood pressure; NYHA, new york heart association classification to stages of heart failure.

All patients with events were already in NYHA class II-III, but only 66.67% of the event-free group were symptomatic. More patients in the event group had pulmonary rales, whereas the presence of peripheral oedema was not different.

Echocardiographic parameters related to the severity of the valvular disease significantly differed between the two groups (Table 2) LVEF was significantly worse in the event group. Parameters describing right ventricular (RV) function also significantly differed: the pulmonary artery systolic pressure (PASP) was higher, and the tricuspid annular plane systolic excursion (TAPSE) was lower in the event group. RV-pulmonary artery (PA) coupling, expressed by TAPSE/PASP ratio, was also significantly different in patients with and without events (Table 2).

**TABLE 2:** Baseline echocardiographic characteristics of the study population and comparisons between patients with and without events.

	All Patients (*n* = 75)	Event-free Group (*n* = 47)	HF Event Group (*n* = 28)	*p*
Peak Ao Gradient (mmHg)	59.61 ± 22	54.74 ± 19.3	67.79 ± 24	0.012
Mean Ao Gradient (mmHg)	37.60 ± 13.4	34.45 ± 12.6	42.89 ± 13.2	0.008
AVA (cm^2^)	0.78 ± 0.2	0.83 ± 0.3	0.71 ± 0.2	0.068
LAVI (ml/m^2^)	34.23 ± 19.5	35.34 ± 17.1	44.64 ± 21.5	0.054
LASr (%)	23.55 ± 12.7	24.93 ± 12.9	16.22 ± 9.5	0.173
LA stiffness	0.75 ± 1.1	0.57 ± 0.4	1.01 ± 0.9	0.424
EDV (ml)	114.80 ± 29	115.40 ± 27.5	113.57 ± 32.5	0.806
ESV (ml)	40.97 ± 20.4	36.83 ± 15.8	49.43 ± 26	0.041
EF (Simpson) %	63.32 ± 10.6	67.67 ± 7.4	56.02 ± 11.2	<0.001
IVS (mm)	12.33 ± 1.5	12.11 ± 1.2	12.71 ± 1.9	0.144
PW (mm)	11.97 ± 1.3	11.94 ± 1.2	12.04 ± 1.5	0.762
LV GLS (%)	−17.03 ± 8.5	−17.08 ± 9.8	−16.90 ± 4.3	0.954
PASP (mmHg)	36.59 ± 15.7	31.00 ± 11.5	45.79 ± 17.4	<0.001
E (cm/s)	83.21 ± 31.6	81.46 ± 28.3	85.69 ± 36.1	0.605
A (cm/s)	98.86 ± 27.5	105.44 ± 28.1	88.57 ± 23.7	0.020
E/A	0.87 ± 0.4	0.78 ± 0.2	1.00 ± 0.5	0.093
DCT (ms)	229.29 ± 63.5	238.79 ± 64.2	215.05 ± 60.4	0.177
E’ (cm/s)	8.55 ± 3.5	8.72 ± 3.1	8.27 ± 4.2	0.668
E/e' (cm/s)	11.35 ± 6.2	10.83 ± 5.2	12.17 ± 7.6	0.457
RV Basal diameter (mm)	35.55 ± 4.1	34.77 ± 3.3	36.44 ± 4.7	0.125
TAPSE (mm)	23.20 ± 4.9	24.54 ± 4.7	21.25 ± 4.6	0.006
RV-PA coupling	0.74 ± 0.35	0.87 ± 0.33	0.55 ± 0.29	*p* < 0.001
Lung ultrasound			
Total number of B-lines (n)	22 ± 22	18 ± 23	29 ± 18	0.028
≥15 B-lines	38 (50.6%)	17 (36.1%)	21 (75%)	0.001
≥30 B-lines	22 (29.3%)	8 (17.%)	14 (50%)	0.002

Data are expressed as mean±SD, or number and percentage.

Ao Peak Gradient: estimated peak pressure gradient across the aortic valve, Ao mean gradient: estimated mean gradient across the aortic valve, AVA: calculated aortic valve area, LV EF: left ventricular Ejection Fraction, LV GLS: left ventricular Global Longitudinal Strain, IVS: intraventricular septum thickness, PW: posterior wall thickness, LAVI: left atrial volume index, LASR: left atrial reservoir strain, LA, stiffness: left atrial stiffness, E: early mitral inflow peak velocity, A: late mitral peak inflow velocity, DCT: E wave deceleration time, E/E’mean: the relationship between maximal values of passive mitral inflow (E, PW-Doppler) and lateral early diastolic mitral annular velocities (E′ TDI) PASP: pulmonary artery systolic pressure, TAPSE: Tricuspid annular plane systolic excursion. RV-PA, coupling is the ratio of TAPSE, and PASP.

**FIGURE 1:**
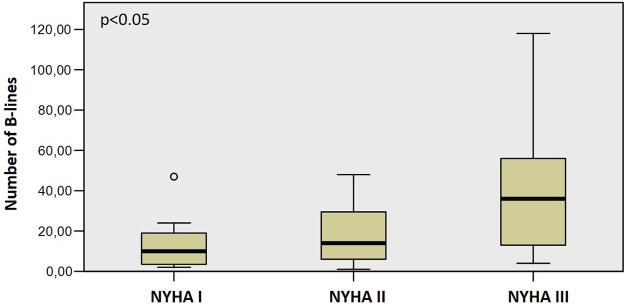
The increasing number of B-lines with worsening NYHA functional class.

We found a severe degree of B-lines (≥30) in 29.33% of all patients. LUS also was different, with more B-lines in the event group (*p* = 0.028) and more patients with a severe degree of B-lines (≥30 B-lines, *p* = 0.002). The number of B-lines increased significantly along with the worsening NYHA functional classes (Figure 1), from 13 ± 12 in NYHA Class I, through 19 ± 15 in Class II, to 43 ± 34 in Class III (*p* < 0.05, rho = 0.383). Patients with severe AS had significantly more B-lines than patients with moderate AS (14 ± 13 vs. 25 ± 24, *p* < 0.05).

**FIGURE 2:**
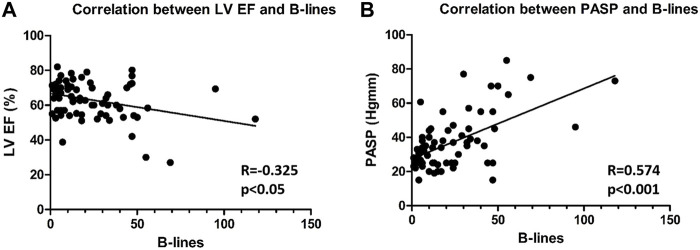
Correlation between B-lines and LVEF **(A)** and PASP **(B)** (LVEF: Left ventricular ejection fraction, PASP: Pulmonary arterial systolic pressure).

We also found that the number of B-lines was correlated (Figures 2A,B) with LVEF (R = −0.325, *p* < 0.05) and PASP (R = 0.574, *p* < 0.001). We did not find a significant correlation between E/e' and B-lines or LAVI and B-lines. Having ≥ 30 B-lines significantly increased the risk of endpoint events [(hazard ratio B-lines CI: 2.79 (1.03–7.54), *p* < 0.05)]. During multivariable modelling, B-lines and mean aortic gradient were the independent predictors of events. Univariate and multivariate predictors of the different endpoints are reported in Table 3.

**TABLE 3:** Cox regression analysis.

	Univariate Analysis		Multivariate Analysis	
	Hr (95% CI)	*p* value	Hr (95% CI)	*p* value
Age	1.06 (1.01−1.11)	0.018	1.03 (0.98−1.08)	0.271
Ao Mean Gradient	1.04 (1.02−1.07)	<0.001	1.04 (1.01−1.07)	0.004
PASP	1.04 (1.02−1.06)	<0.001	1.01 (0.98−1.04)	0.456
B-lines ≥15	2.609 (1.10−6.19)	0.029	—	—
B-lines ≥30	2.86 (1.36−6.03)	0.006	2.79 (1.03−7.54)	0.043

**FIGURE 3:**
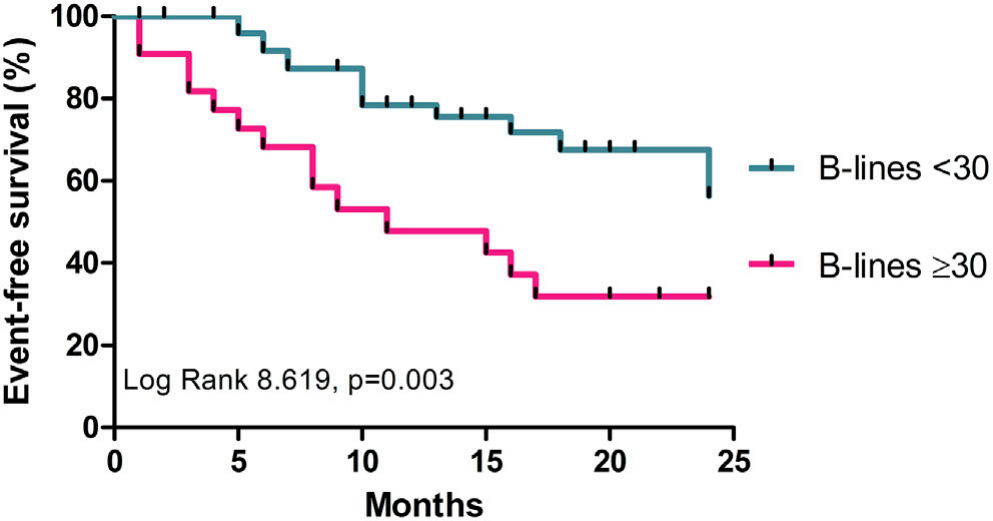
Comparison of HF endpoints among patients with≥30 and <30 B-lines.

The event-free survival was significantly worse among those who had equal or more than 30 B-lines (Log Rank 8.619, *p* = 0.003) (Figure 3).

## Discussion

To the best of our knowledge, this is the first study to address the prognostic value of B-lines for the prediction of adverse events in patients with AS. Our results show that the assessment of B-lines in AS adequately reflects patients functional class and the haemodynamic consequences caused by AS. Presence of severe B-lines (≥30) strongly predicts adverse events.

Current guidelines advise valve replacement when an integrative evaluation of pressure gradients, AVA, the extent of valve calcification, and flow indicates severe AS, and there is evidence of LV decompensation evaluated by echocardiographic measurements or appearance of symptoms (Vahanian et al., 2021). The guidelines also pointed out some additional prognostic markers, which also help decide AVR (Vahanian et al., 2021). Exercise stress echocardiography may provide prognostic information in asymptomatic patients (Marechaux et al., 2010); cardiac magnetic resonance enables to assess myocardial fibrosis (Azevedo et al., 2010). Moreover, natriuretic peptides have been shown to predict symptom-free survival and outcome in normal and low-flow severe AS (Bergler-Klein et al., 2004). These predictors especially stress echocardiography and cardiac magnetic resonance, are not always available, and the repeated measurements are not feasible.

LV hypertrophy is a mechanism of accommodation in AS to restore wall stress and maintain cardiac output under increasing pressure afterload caused by the stenotic valve. However, the progressive cardiomyocyte death and consequent fibrosis that accompanies LV hypertrophy may lead to the development of LV systolic and diastolic dysfunction and finally to HF. Historical data have shown that the time from the onset of symptoms to death is about 2 years in patients who develop HF (Frank et al., 1973). Besides the prognostic importance of HF in recent years, the data supports that cardiac damage also holds prognostic significance after AVR (Généreux et al., 2017). Stages of cardiac damage in patients with severe AS have been defined from stage 1 to stage 4. These are: LV dysfunction, left atrial enlargement, pulmonary hypertension, and RV dysfunction. Each stage is associated with an increased mortality risk within one year, ranging from 4% in stage 0 (no damage) up to 25% in stage 4 (Généreux et al., 2017). Our results are consistent with these data, showing that patients with HF events have lower EF, lower TAPSE, higher PASP. However, the worsening LVEF is a late and insensitive marker of myocardial dysfunction (Généreux et al., 2017).

LV systolic and diastolic dysfunction and mitral regurgitation result in PC, which is a common finding in patients with HF. LUS assessment of PC by B-lines has been demonstrated to be an excellent diagnostic tool (Pellicori et al., 2019) (Lichtenstein et al., 1997; Jambrik et al., 2004; Picano et al., 2010; Pivetta et al., 2015). The quick examination time with 100% feasibility allows this method to be easily performed during bedside patient evaluation. Decompensation is clinically silent in most patients and is often not recognized until developing rapid progression that requires urgent hospitalization. LUS can assess lung oedema noninvasively in real-time, even at an early subclinical stage. B-lines are helpful for the differential diagnosis of acute HF syndromes from non-cardiac causes of acute dyspnoea in the emergency setting, with high sensitivity and specificity (Al Deeb et al., 2014) The number B-lines measured by LUS correlated well with NT-proBNP level (Gargani et al., 2008; Miglioranza et al., 2013) (Volpicelli et al., 2008) and the indexes of diastolic dysfunction (Gargani, 2011). (Gargani et al., 2008). (Frassi et al., 2007a). We did not find a significant correlation between E/e' or LAVI and B-lines. Previous studies have shown this correlation, especially when assessing B-lines during exercise. However, this relation has never been tested in patients with significant aortic stenosis. Reddy and colleagues simultaneously performed stress tests, lung ultrasound, and right heart catheterization in HFpEF. B-lines increase during exercise was associated with lower RV systolic velocity and RV fractional area change, worse RV-PA coupling, higher pulmonary capillary wedge pressure (PCWP), and higher pulmonary artery (PA) pressure. However, baseline E/e’ was not higher in patients who increased B-lines during exercise). (Reddy et al., 2019). Simonovic D et al. enrolled HFpEF patients and performed exercise stress echocardiography and B-lines assessment; again, the resting E/e’ value was not higher in patients with ≥10 B-lines at exercise (Simonovic et al., 2021). Hubert and colleagues performed direct measurements of LV filling pressure and B-lines assessment on patients with different cardiovascular conditions, undergoing coronary angiography, and found that the total number of B-lines was significantly higher in the elevated LVEDP group (≥20 mmHg). They underline that the diagnostic capacity of B-lines to identify elevated LVEDP is higher than that of classical echocardiographic strategies (Hubert et al., 2019). Volpicelli et al. also assessed B-lines in critically ill patients with simultaneous PCWP monitoring (with only 10 patients with cardiogenic pulmonary edema), confirming that B-lines allow good prediction of pulmonary congestion indicated by EVLW. Whereas B-lines assessment is of limited usefulness for the prediction of hemodynamic congestion indicated by PCWP (Volpicelli et al., 2014). Indeed the added value of B-lines is being a sign of pulmonary congestion, independent of the degree of hemodynamic congestion. Platz et al. also investigated patients with unexplained dyspnea with invasive hemodynamic measurements and LUS: the number of B-lines at rest was correlated to PCWP and mean pulmonary artery pressure (Platz et al., 2019). Reddy et al. showed that stress-induced B-lines elevation was mainly dependent on RV dysfunction and pulmonary hemodynamics (Reddy et al., 2019). We also found a significant correlation between B-lines and RV-PA coupling, expressed by TAPSE/PASP ratio (r −0.443, *p* < 0.001). The meta-analysis by Kobayashi et al. also confirmed that worse RV function and RV–PA coupling were associated with higher B-line counts on admission and at discharge regardless of LVEF (Kobayashi et al., 2021). B-lines also have an exceptional prognostic value, shown in patients with HF (Frassi et al., 2007b; Gargani et al., 2015; Platz et al., 2016; Miglioranza et al., 2017; Gargani et al., 2021). The predictive value is independent and additive over conventional clinical, imaging, and laboratory markers, such as a functional class, signs of congestion, namely crackles over the lungs, LVEF, pulmonary artery systolic pressure, or cardiac natriuretic peptide levels (Frassi et al., 2007b; Bedetti et al., 2010; Gustafsson et al., 2015; Platz et al., 2016). Our results are consistent with these previous findings: patients with AS-related PC have significantly more B-lines, and patients with ≥30 B-lines have significantly more HF-related events and death. According to previous studies, we chose ≥30 B-lines in 28 scanning sites to determine severe congestion (Miglioranza et al., 2017) (Coiro et al., 2015). Residual pulmonary congestion of ≥30 B-lines at discharge in patients with acute heart failure, irrespectively of the HF etiology and EF, is a strong and independent predictor of outcome (Coiro et al., 2015). The determination of B-lines in AS is a promising method because establishing symptomatic status in this population is challenging due to their usually sedentary lifestyle and high prevalence of co-morbidities (Chin et al., 2015), as ageing and concomitant medical problems can cause symptoms similar to AS or conceal them by restricting physical activity. Even though angina and syncope are easily detectable symptoms, HF can be indolent. Therefore, there is a rationale for using additional methods to detect HF early.

Several attempts were made to improve the prognostic stratification of AS patients. CAIMAN-ECHO score is an echocardiography based tool for asymptomatic, moderate or severe AS patients. It takes into account the calcium score of the aortic valve, inappropriate LV mass, and peak gradient across the aortic valve to predict the risk of cardiovascular events (all-cause mortality, AVR, hospitalization for MI and HF) (Cioffi et al., 2013). Monin et al. developed a scoring system for patients with asymptomatic severe AS, including gender, BNP level, and peak aortic jet velocity. It can be used for the prediction of midterm risk of death and AVR (Monin et al., 2009).Kearney et al. followed up AS patients older than 60 years of age (mild to severe valvular disease) for 18 years, and he found that age-adjusted Charlson co-morbidity index and grade of LV dysfunction were risk factors of all-cause mortality while having an AVR acted as a protective factor (Kearney et al., 2012). The predictive role of apical rotation was also assessed in a group of patients with symptomatic and asymptomatic severe AS and preserved EF. It was found that increased apical rotation was linked to worse survival (Holmes et al., 2015). It was also found that raised BNP and troponin I are also markers of adverse prognosis in asymptomatic patients with moderate-to-severe asymptomatic AS (Clavel et al., 2014) (Chin et al., 2014).

The assessment of B-lines has several advantages in patients with moderate and severe AS. The expansion of regular, standard TTE by LUS should improve risk stratification of patients. Cardiac damage, especially LV, mitral valve and LA dysfunction, results in PC and, consequently in HF signs and symptoms. Hence, early detection by LUS holds incremental prognostic and diagnostic possibilities. A more accurate PC assessment might optimize the timing of valve surgery or help tailor HF therapy. It may identify high-risk AS patients whose concomitant heart disease aggravates PC, for example, ischemic LV dysfunction, cardiomyopathies, and mitral valve disease. B-line assessment before surgery (TAVR or open-heart surgery) may influence postoperative events; however, further studies are needed to confirm these hypotheses.

## Limitations

The sample size is relatively small, and our series may not represent the average patient with moderate or severe AS. The detection of B-lines does not necessarily imply their cardiogenic origin; however, we applied strict criteria to exclude patients with potential non-cardiogenic B-lines. We did not collect the baseline levels of NT-proBNP.

## Conclusion

In a moderate and severe AS, B-lines evaluated by LUS are significantly correlated with NYHA functional class, LV ejection fraction, and pulmonary artery systolic pressure. During the short-term follow-up, a higher number of B-lines was associated with HF-related adverse events and death. Given its accuracy and simplicity, LUS could be considered a reliable tool for assessing PC in patients with AS, and could be incorporated as an extension of the physical examination.

## Data Availability

The raw data supporting the conclusion of this article will be made available by the authors, without undue reservation.
